# Supplementary material for: De novo synthesized Min proteins drive oscillatory liposome deformation and regulate FtsA-FtsZ cytoskeletal patterns
Source: Nat Commun. 2019 Oct 31;10:4969. doi: 10.1038/s41467-019-12932-w (PMC6823393; doi:10.1038/s41467-019-12932-w)
Supplement: Supplementary file 1 — Supplementary Information [file 41467_2019_12932_MOESM1_ESM.pdf]

Supplementary information for

De novo synthesized Min proteins drive oscillatory liposome deformation and regulate FtsA-FtsZ cytoskeletal patterns

by Godino et al.

SUPPLEMENTARY METHODS

Sequence of MinD construct

TAATACGACTCACTATAGGGGAATTGTGAGCGGATAACAATTCCCCTCTAGAAATAATTTTGTTTAACTTTAAGAAGGAGATATAC

ATatgGCACGCATTATTGTTGTTACTTCGGGCAAAGGGGGTGTTGGTAAGACAACCTCCAGCGCGGCCATCGCCACTGGTTTGGCC

CAGAAGGGAAAGAAAACTGTCGTGATAGATTTTGATATCGGCCTGCGTAATCTCGACCTGATTATGGGTTGTGAACGCCGGGTCG

TTTACGATTTCGTCAACGTCATTCAGGGCGATGCAACGCTAAATCAGGCGTTAATTAAAGATAAGCGTACTGAAAATCTCTATATTC

TGCCGGCATCGCAAACACGCGATAAAGATGCCCTCACCCGTGAAGGGGTCGCCAAAGTTCTTGATGATCTGAAAGCGATGGATTT

TGAATTTATCGTTTGTGACTCCCCGGCAGGGATTGAAACCGGTGCGTTAATGGCACTCTATTTTGCAGACGAAGCCATTATTACCAC

CAACCCGGAAGTCTCCTCAGTACGCGACTCTGACCGTATTTTAGGCATTCTGGCGTCGAAATCACGCCGCGCAGAAAATGGCGAA

GAGCCTATTAAAGAGCACCTGCTGTTAACGCGCTATAACCCAGGCCGCGTAAGCAGAGGTGACATGCTGAGCATGGAAGATGTGC

TGGAGATCCTGCGCATCAAACTCGTCGGCGTGATCCCAGAGGATCAATCAGTATTGCGCGCCTCTAACCAGGGTGAACCGGTCATT

CTCGACATTAACGCCGATGCGGGTAAAGCCTACGCAGATACCGTAGAACGTCTGTTGGGAGAAGAACGTCCTTTCCGCTTCATTGA

AGAAGAGAAGAAAGGCTTCCTCAAACGCTTGTTCGGAGGAtaaGGATCCGGCTGCTAACAAAGCCCGAAAGGAAGCTGAGTTGGC

TGCTGCCACCGCTGAGCAATAACTAGCATAACCCCTTGGGGCCTCTAAACGGGTCTTGAGGGGTTTTTTG

Sequence of optimized MinE construct

TAATACGACTCACTATAGGGGAATTGTGAGCGGATAACAATTCCCCTCTAGAAATAATTTTGTTTAACTTTAAGAAGGAGATATAC

ATatgGCGCTGCTGGATTTCTTTCTGAGCCGTAAGAAAAACACCGCGAACATCGCGAAAGAGCGTCTGCAAATCATTGTTGCGGAG

CGTCGTCGTAGCGATGCGGAACCGCACTACCTGCCGCAGCTGCGTAAAGATATCCTGGAAGTGATTTGCAAGTATGTTCAAATTGA

CCCGGAGATGGTGACCGTTCAGCTGGAACAAAAGGACGGTGATATCAGCATTCTGGAGCTGAACGTTACCCTGCCGGAAGCGGA

GGAACTGAAGtaaGGATCCGGCTGCTAACAAAGCCCGAAAGGAAGCTGAGTTGGCTGCTGCCACCGCTGAGCAATAACTAGCATA

ACCCCTTGGGGCCTCTAAACGGGTCTTGAGGGGTTTTTTG

Sequence of optimized MinC construct

TAATACGACTCACTATAGGGGAATTGTGAGCGGATAACAATTCCCCTCTAGAAATAATTTTGTTTAACTTTAAGAAGGAGATATAC

ATATGAGCAATACCCCGATTGAACTGAAAGGCAGCAGCTTTACCCTGAGCGTTGTGCATCTGCATGAAGCGGAGCCGAAGGTGAT

CCATCAGGCGCTGGAGGACAAAATCGCGCAAGCGCCGGCGTTCCTGAAGCATGCGCCGGTGGTTCTGAACGTGAGCGCGCTGGA

AGATCCGGTTAACTGGAGCGCGATGCACAAAGCGGTGAGCGCGACCGGTCTGCGTGTGATTGGTGTTAGCGGCTGCAAGGACGC

GCAGCTGAAAGCGGAGATCGAAAAGATGGGTCTGCCGATTCTGACCGAAGGCAAGGAAAAAGCGCCGCGTCCGGCGCCGACCCC

GCAGGCGCCGGCGCAAAACACCACCCCGGTGACCAAGACCCGTCTGATCGACACCCCGGTTCGTAGCGGCCAGCGTATCTACGCG

CCGCAATGCGATCTGATTGTGACCAGCCACGTTAGCGCGGGTGCGGAGCTGATTGCGGATGGTAACATTCACGTGTATGGTATGA

TGCGTGGCCGTGCGCTGGCGGGTGCGAGCGGCGATCGTGAAACCCAGATTTTCTGCACCAACCTGATGGCGGAGCTGGTTAGCAT

CGCGGGTGAATACTGGCTGAGCGATCAAATTCCGGCGGAGTTTTATGGTAAGGCGGCGCGTCTGCAACTGGTGGAGAATGCGCT

GACCGTGCAACCGCTGAACTAAGGATCCGGCTGCTAACAAAGCCCGAAAGGAAGCTGAGTTGGCTGCTGCCACCGCTGAGCAATA

ACTAGCATAACCCCTTGGGGCCTCTAAACGGGTCTTGAGGGGTTTTTTG

Sequence of optimized FtsA construct

TAATACGACTCACTATAGGGGAATTGTGAGCGGATAACAATTCCCCTCTAGAAATAATTTTGTTTAACTTTAAGAAGGAGATATAC

ATATGATCAAGGCGACCGACCGTAAGCTGGTTGTGGGCCTGGAGATTGGCACCGCGAAGGTTGCGGCGCTGGTTGGCGAGGTTC

TGCCGGATGGTATGGTTAACATTATCGGCGTTGGTAGCTGCCCGAGCCGTGGCATGGACAAAGGTGGTGTGAACGACCTGGAAA

GCGTGGTTAAGTGCGTGCAGCGTGCGATTGACCAGGCGGAGCTGATGGCGGACTGCCAAATCAGCAGCGTTTACCTGGCGCTGA

GCGGCAAGCACATCAGCTGCCAAAACGAGATTGGTATGGTGCCGATTAGCGAAGAGGAAGTTACCCAGGAAGATGTGGAGAACG

TGGTTCACACCGCGAAAAGCGTTCGTGTGCGTGATGAACACCGTGTGCTGCACGTTATCCCGCAAGAATACGCGATCGATTACCA

GGAAGGTATCAAAAACCCGGTTGGTCTGAGCGGTGTTCGTATGCAGGCGAAAGTGCACCTGATTACCTGCCACAACGATATGGCG

AAGAACATTGTGAAAGCGGTTGAACGTTGCGGTCTGAAGGTTGACCAGCTGATCTTCGCGGGTCTGGCGAGCAGCTACAGCGTTC

TGACCGAAGATGAGCGTGAACTGGGTGTTTGCGTTGTGGATATCGGCGGTGGCACGATGGATATCGCGGTGTATACCGGTGGCG

CGCTGCGTCACACCAAAGTGATTCCGTATGCGGGTAACGTGGTTACCAGCGACATCGCGTACGCGTTTGGCACCCCGCCGAGCGA

TGCGGAGGCGATCAAAGTGCGTCACGGTTGCGCGCTGGGTAGCATTGTGGGTAAAGATGAGAGCGTGGAAGTTCCGAGCGTTGG

TGGCCGTCCGCCGCGTAGCCTGCAACGTCAGACCCTGGCGGAAGTTATCGAGCCGCGTTACACCGAACTGCTGAACCTGGTGAAC

GAAGAGATCCTGCAACTGCAAGAGAAACTGCGTCAGCAAGGTGTTAAGCACCACCTGGCGGCGGGCATTGTTCTGACCGGCGGT

GCGGCGCAGATCGAAGGTCTGGCGGCGTGCGCGCAACGTGTTTTCCACACCCAAGTTCGTATCGGTGCGCCGCTGAACATCACCG

GTCTGACCGATTACGCGCAAGAGCCGTACTATAGCACCGCGGTTGGTCTGCTGCACTATGGCAAAGAGAGCCACCTGAACGGCGA

GGCGGAAGTGGAGAAGCGTGTGACCGCGAGCGTTGGTAGCTGGATTAAGCGTCTGAATAGCTGGCTGCGTAAGGAGTTCTAAGG

ATCCGGCTGCTAACAAAGCCCGAAAGGAAGCTGAGTTGGCTGCTGCCACCGCTGAGCAATAACTAGCATAACCCCTTGGGGCCTC

TAAACGGGTCTTGAGGGGTTTTTTG

SUPPLEMENTARY TABLES

Protein	Peptide	Precursor Ion	MS1 Res	Product Ion	MS2 Res	Fragmentor (V)	Collision Energy (eV)	Cell Accelerator Voltage (V)	Ret Time (min)	Delta Ret Time	Ion Name
MinD	IIVVTSGK	408.763103	Unit	590.350802	Unit	130	13.7	4	5.07	1.5	y6
MinD	IIVVTSGK	408.763103	Unit	491.282388	Unit	130	13.7	4	5.07	1.5	y5
MinD	IIVVTSGK	408.763103	Unit	392.213974	Unit	130	13.7	4	5.07	1.5	y4
MinD	IIVVTSGK	408.763103	Unit	227.175404	Unit	130	13.7	4	5.07	1.5	b2
MinD	TENLYILPASQTR	753.401555	Unit	1048.578571	Unit	130	24.4	4	11.62	1.5	y9
MinD	TENLYILPASQTR	753.401555	Unit	885.515242	Unit	130	24.4	4	11.62	1.5	y8
MinD	TENLYILPASQTR	753.401555	Unit	772.431178	Unit	130	24.4	4	11.62	1.5	y7
MinD	TENLYILPASQTR	753.401555	Unit	659.347114	Unit	130	24.4	4	11.62	1.5	y6
MinD	TENLYILPASQTR	753.401555	Unit	345.140475	Unit	130	24.4	4	11.62	1.5	b3
MinD	ILGILASK	407.773471	Unit	701.455602	Unit	130	13.6	4	10.32	1.5	y7
MinD	ILGILASK	407.773471	Unit	588.371538	Unit	130	13.6	4	10.32	1.5	y6
MinD	ILGILASK	407.773471	Unit	418.26601	Unit	130	13.6	4	10.32	1.5	y4
MinD	ILGILASK	407.773471	Unit	227.175404	Unit	130	13.6	4	10.32	1.5	b2
MinD	LVGVIPEDQSVLR	712.909016	Unit	943.484336	Unit	130	23.1	4	13.19	1.5	y8
MinD	LVGVIPEDQSVLR	712.909016	Unit	270.181218	Unit	130	23.1	4	13.19	1.5	b3
MinD	LVGVIPEDQSVLR	712.909016	Unit	369.249632	Unit	130	23.1	4	13.19	1.5	b4
MinD	LVGVIPEDQSVLR	712.909016	Unit	482.333696	Unit	130	23.1	4	13.19	1.5	b5
MinD	AYADTVER	462.724706	Unit	690.341694	Unit	130	15.3	4	3.76	1.5	y6
MinD	AYADTVER	462.724706	Unit	619.30458	Unit	130	15.3	4	3.76	1.5	y5
MinD	AYADTVER	462.724706	Unit	504.277637	Unit	130	15.3	4	3.76	1.5	y4
MinD	AYADTVER	462.724706	Unit	235.107718	Unit	130	15.3	4	3.76	1.5	b2
MinE	LQIIVAER	471.292559	Unit	700.435201	Unit	130	15.6	4	9.18	1.5	y6
MinE	LQIIVAER	471.292559	Unit	587.351137	Unit	130	15.6	4	9.18	1.5	y5
MinE	LQIIVAER	471.292559	Unit	474.267073	Unit	130	15.6	4	9.18	1.5	y4
MinE	LQIIVAER	471.292559	Unit	242.149918	Unit	130	15.6	4	9.18	1.5	b2
MinE	LQIIVAER	471.292559	Unit	355.233982	Unit	130	15.6	4	9.18	1.5	b3
MinE	DILEVIC[+57.021464]K	495.270435	Unit	761.422587	Unit	130	16.4	4	13.24	1.5	y6
MinE	DILEVIC[+57.021464]K	495.270435	Unit	648.338523	Unit	130	16.4	4	13.24	1.5	y5
MinE	DILEVIC[+57.021464]K	495.270435	Unit	519.29593	Unit	130	16.4	4	13.24	1.5	y4
MinE	DILEVIC[+57.021464]K	495.270435	Unit	307.143452	Unit	130	16.4	4	13.24	1.5	y2
MinE	DILEVIC[+57.021464]K	495.270435	Unit	229.118283	Unit	130	16.4	4	13.24	1.5	b2
MinE	YVQIDPEMVTVQLEQK	960.492784	Unit	1416.703907	Unit	130	30.8	4	16.3	1.5	y12
MinE	YVQIDPEMVTVQLEQK	960.492784	Unit	1301.676964	Unit	130	30.8	4	16.3	1.5	y11
MinE	YVQIDPEMVTVQLEQK	960.492784	Unit	845.472708	Unit	130	30.8	4	16.3	1.5	y7
MinE	YVQIDPEMVTVQLEQK	960.492784	Unit	263.139019	Unit	130	30.8	4	16.3	1.5	b2
MinE	YVQIDPEMVTVQLEQK	960.492784	Unit	391.197596	Unit	130	30.8	4	16.3	1.5	b3
MinE	DGDISILELNVTLPEAEELK	1099.575556	Unit	1242.657609	Unit	130	35.1	4	21.53	1.5	y11
MinE	DGDISILELNVTLPEAEELK	1099.575556	Unit	1029.546267	Unit	130	35.1	4	21.53	1.5	y9
MinE	DGDISILELNVTLPEAEELK	1099.575556	Unit	928.498589	Unit	130	35.1	4	21.53	1.5	y8
MinE	DGDISILELNVTLPEAEELK	1099.575556	Unit	815.414525	Unit	130	35.1	4	21.53	1.5	y7

MinC	IAQAPAFLK	479.789652	Unit	774.450851	Unit	130	8.9	4	8.81	3	y7
MinC	IAQAPAFLK	479.789652	Unit	646.392273	Unit	130	8.9	4	8.81	3	y6
MinC	IAQAPAFLK	479.789652	Unit	575.355159	Unit	130	8.9	4	8.81	3	y5
MinC	IAQAPAFLK	479.789652	Unit	185.128454	Unit	130	8.9	4	8.81	3	b2
MinC	IAQAPAFLK	479.789652	Unit	384.224145	Unit	130	8.9	4	8.81	3	b4
MinC	MGLPILTEGK	529.79936	Unit	927.550959	Unit	130	11.5	4	12.53	3	y9
MinC	MGLPILTEGK	529.79936	Unit	870.529495	Unit	130	11.5	4	12.53	3	y8
MinC	MGLPILTEGK	529.79936	Unit	302.153288	Unit	130	11.5	4	12.53	3	b3
MinC	MGLPILTEGK	529.79936	Unit	625.37418	Unit	130	11.5	4	12.53	3	b6
EF-Tu	TTLTAAITTVLAK	652.39521	Unit	887.556044	Unit	130	21.2	4	16.34	1.5	y9
EF-Tu	TTLTAAITTVLAK	652.39521	Unit	816.51893	Unit	130	21.2	4	16.34	1.5	y8
EF-Tu	TTLTAAITTVLAK	652.39521	Unit	745.481817	Unit	130	21.2	4	16.34	1.5	y7
EF-Tu	TTLTAAITTVLAK	652.39521	Unit	632.397753	Unit	130	21.2	4	16.34	1.5	y6
EF-Tu	GITINTSHVEYDTPTR	601.96724	Unit	752.357344	Unit	130	16.9	4	9.26	1.5	y6
EF-Tu	GITINTSHVEYDTPTR	601.96724	Unit	474.267073	Unit	130	16.9	4	9.26	1.5	y4
EF-Tu	GITINTSHVEYDTPTR	601.96724	Unit	710.328587	Unit	130	16.9	4	9.26	1.5	y12
EF-Tu	GITINTSHVEYDTPTR	601.96724	Unit	187.113335	Unit	130	16.9	4	9.26	1.5	y3

Supplementary Table 1: Transitions of the MS/MS measurements used in this study.

	Intercept value	Intercept standard error	Slope value	Slope standard error	R^2 value
**MinD (end-point assay)**					
IIVVTSGK	-1422	894	6773	879	0.935
TENLYILPASQTR	-663	386	5522	217	0.993
ILGILASK	-2337	1878	19090	1235	0.983
LVGVIPEDQSVLR	-1350	717	9949	468	0.991
AYADTVER	-1019	456	4552	333	0.978
**MinD (kinetic assay)**					
IIVVTSGK	-1876	567	7859	329	0.993
TENLYILPASQTR	-4175	1157	11705	593	0.99
ILGILASK	-6153	1794	22846	686	0.996
LVGVIPEDQSVLR	-4883	1479	16642	688	0.993
AYADTVER	-2256	601	7091	329	0.991
**MinE (end-point assay)**					
LQIIVAER	-16171	3717	40112	3533	0.969
DILEVIC[+57]K	-11759	1405	21821	1743	0.974
YVQIDPEMVTVQLEQK	-1235	204	2152	237	0.953
DGDISILELNVTLPEAEELK	-1046	125	1887	173	0.967
**MinE (kinetic assay)**					
LQIIVAER	-13769	7191	36750	4990	0.930
DILEVIC[+57]K	-7882	2820	18262	2406	0.934
YVQIDPEMVTVQLEQK	-620	195	1627	252	0.910
DGDISILELNVTLPEAEELK	-660	353	2080	367	0.886
**MinC (end-point assay)**					
IAQAPAFLK	-501	2080	29228	2236	0.966
MGLPILTEGK	-1820	225	14575	333	0.997

Supplementary Table 2: Fit parameter values for the LC-MS calibration curves shown in Supplementary Fig. 3. The peptide IIVVTSGK of MinD was disregarded for further quantitation because the integrated peak intensity value of the peptide originating from the cell-free synthesized protein was higher than the value obtained with the most concentrated standard. The proteolytic peptides are listed according to their position along the amino acid sequence, i.e. from the most N-terminal (top) to the most C-terminal (bottom) as graphically represented in Fig. 1c for MinD/E and in Supplementary Fig. 4 for MinC. The standard rows for MinD and MinE were repeated on one day for two biological replicates for quantification after 180 min and a second time for a kinetic measurement. On both days, independent standard rows were measured and calculated peak areas were compared only to same-day standards.

Name	Sequence (5' → 3')
ChD382	TCCTTTCGGGCTTTGTTAGCAGCCGGATCCTTATCCTCCGAACAAGCGTTTG
ChD384	TCCTTTCGGGCTTTGTTAGCAGCCGGATCCTTATTTCAGCTCTTCTGCTTCC
ChD511	TTAACTTTAAGAAGGAGATATACATATGGCACGCATTATTGTTG
ChD512	TTAACTTTAAGAAGGAGATATACATATGGCATTACTCGATTTCTTTCTC
ChD709	CAAAAAACCCCTCAAGACCCGTTTAGAGG
ChD757	TAATACGACTCACTATAGGG

Supplementary Table 3: List of primers used in this study

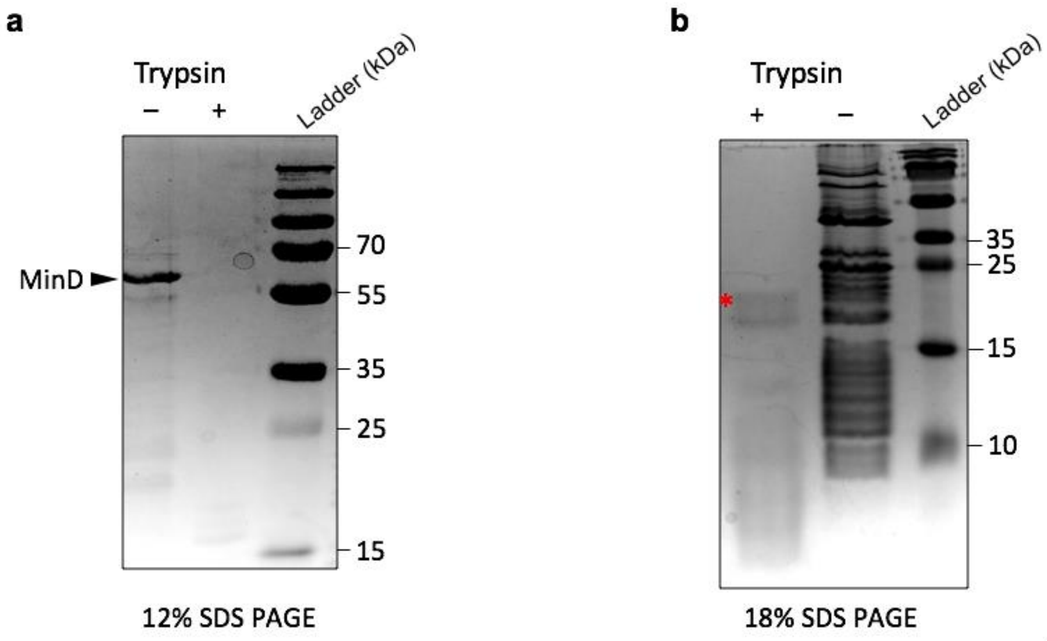

Supplementary Fig. 1: Validation of protein digestion for quantification by LC-MS. a, Coomassie stained SDS PAGE loaded with 1.48 µg of purified MinD and 0.5 µg MinE (equivalent to 10 µM) which were used as a standard for mass spectrometry measurements. The first lane contains the undigested protein sample. The second lane was loaded with a similar sample digested with trypsin (See Methods section). The MinE band is not visible on this gel (below the 15 kDa marker). 5 µl of Pageruler plus ladder was loaded. **b**, Coomassie stained SDS PAGE loaded with PURE*frex*2.0 after 3 h co-expression of the *minD* and *minE* genes. The sample was either loaded directly or after treatment with Trypsin. The star indicates the Trypsin band. 5 µl of Pageruler plus ladder was loaded. Complete protein digestion with both purified MinDE and PURE*frex*2.0 samples was confirmed. Source data are provided as a Source Data file.

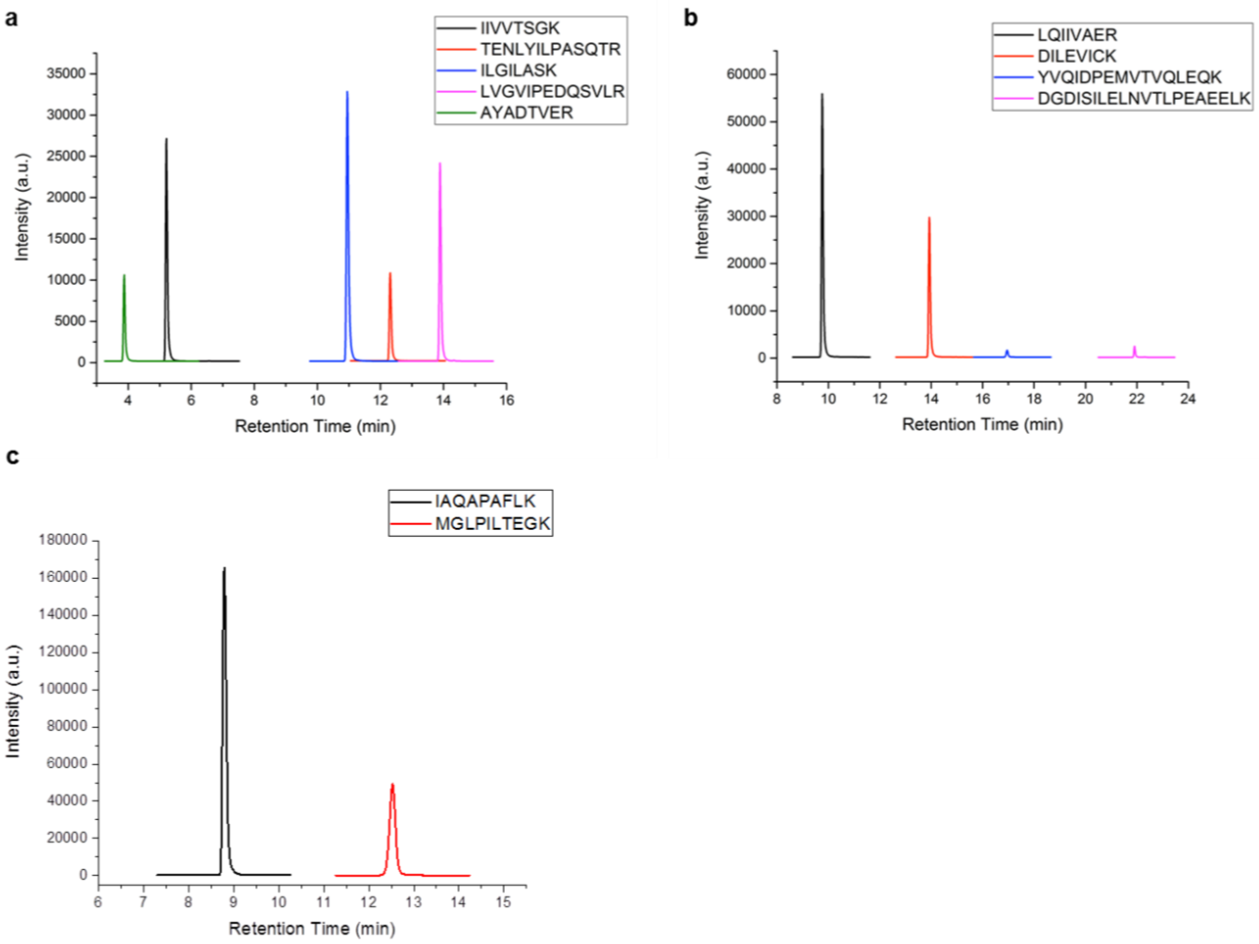

Supplementary Fig. 2: Peptide chromatograms of the LC-MS method. **a**, MinD proteolytic peptides. **b**, MinE proteolytic peptides. **c**, MinC proteolytic peptides.

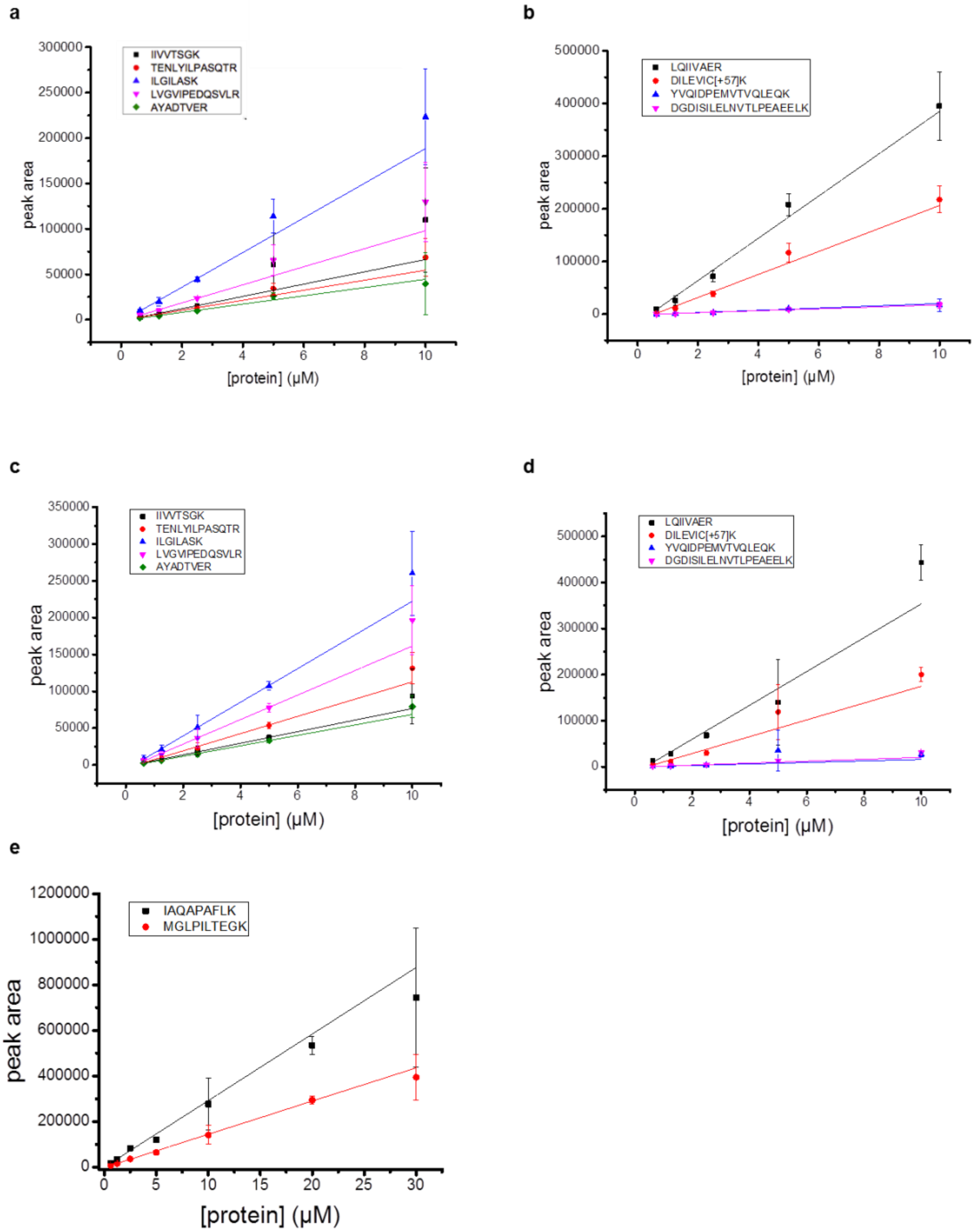

Supplementary Fig. 3: LC-MS calibration curves with MinD and MinE protein standards. The peak area of various proteolytic peptides is plotted versus different concentrations of purified MinD (**a**) and MinE (**b**) for two PURE system reactions incubated for 180 min. Panels (**c**) and (**d**) show a similar calibration for three additional reactions in order to provide kinetic data about MinD and MinE co-expression. **e**, Calibration for the two peptides of MinC. Linear fits through the measurement points are appended for each peptide. Each point represents the average of three independent measurements and the error bars indicate standard deviation. The amino acid sequences of the considered MinD-, MinE- and MinC-specific peptides are displayed in the legend boxes. The extracted fit parameters are reported in Supplementary Table 2. Source data are provided as a Source Data file.

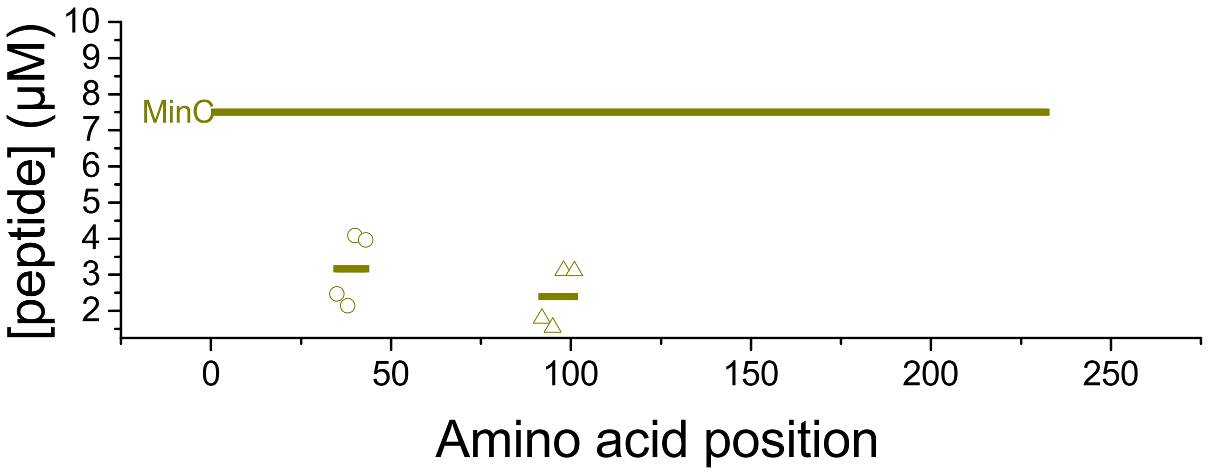

Supplementary Fig. 4: Concentration of MinC proteolytic peptides plotted as a function of their position in the protein primary sequence. The markers represent measurements from four different experiments and the average values are displayed as short segments whose length corresponds to the peptide length. The MinC-annotated line depicts the full length sequence. Source data are provided as a Source Data file.

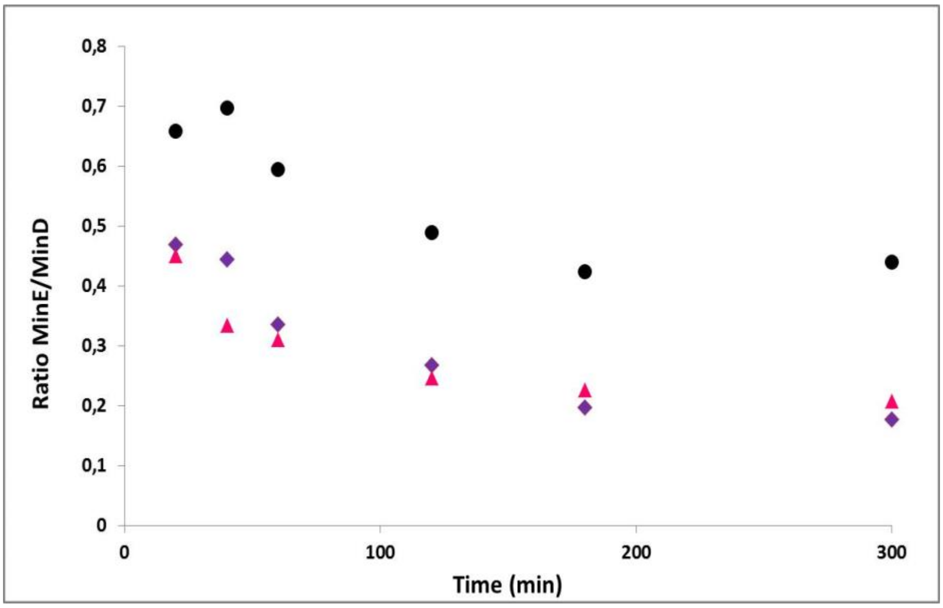

Supplementary Fig. 5: Concentration ratio of MinE and MinD C-terminal peptides in the course of a co-expression reaction. The different markers correspond to data from three independent experiments shown in Fig. 1d.

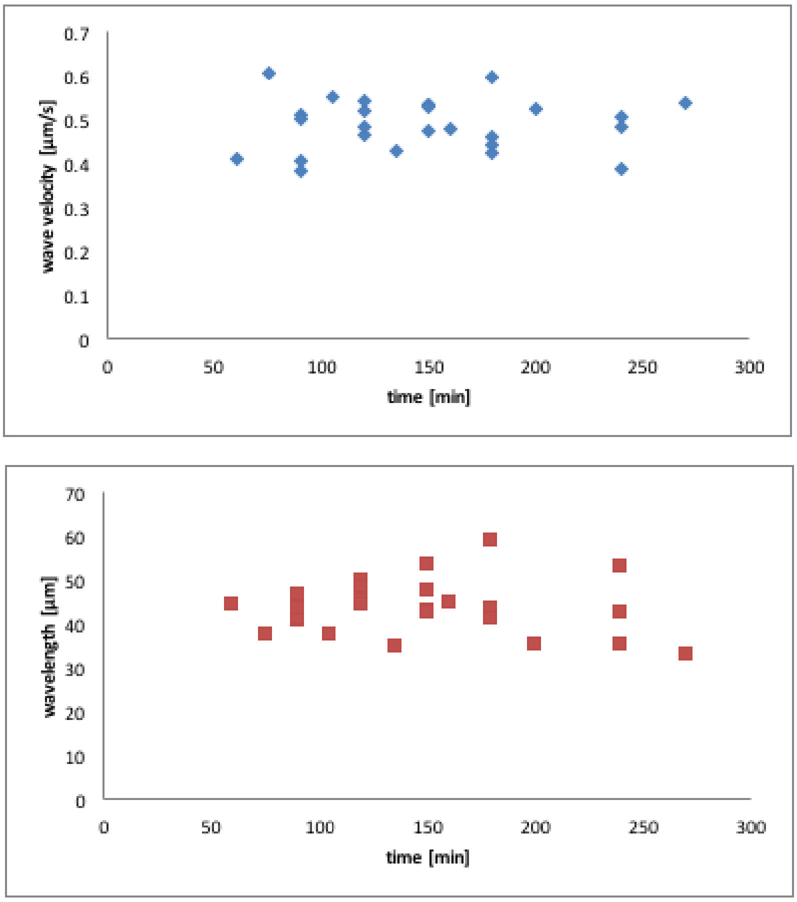

Supplementary Fig. 6: Quantification of Min pattern dynamics from SLB assays with in situ co-expressed MinD/E proteins. Data points are from five different fields of view imaged at different time points, and they have been collected from two independent kinetic experiments.

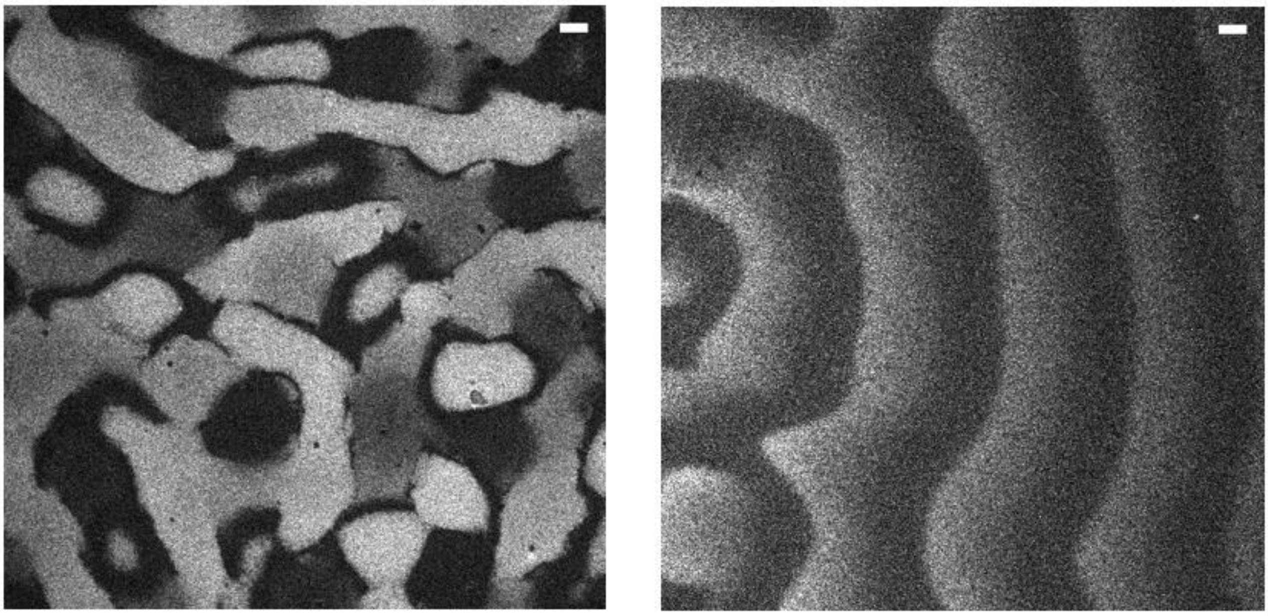

Supplementary Fig. 7: Representative patterns of purified MinD/E proteins mixed in a pre-ran PURE*frex*2.0 solution containing the TP-coding DNA and added on top of an SLB. Standing waves (left panel) and traveling waves (right panel) are observed. MinD and MinE were used at 1 µM each. Traveling waves have a wavelength of ~ 61 µm and a velocity of ~ 0.47 µm s^{-1}. Corresponding videos are shown in Supplementary Movie 3. Scale bars are 10 µm.

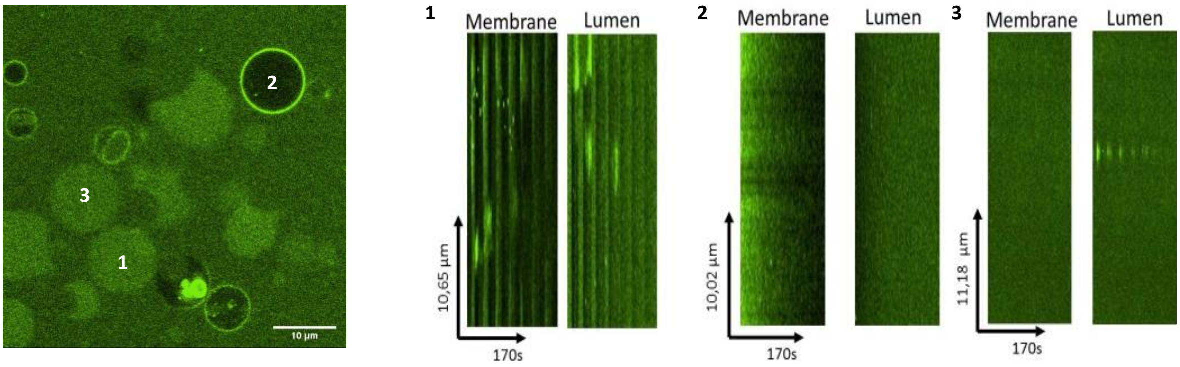

Supplementary Fig. 8: Image analysis of liposomes whose membrane signal attenuates over time while the lumen signal correspondingly increases. Fluorescence microscopy image acquired in the eGFP-MinC channel under similar conditions as shown in Fig. 3 and Fig. 4. The three liposomes analyzed are numbered and their corresponding kymographs are displayed. Liposome 1 exhibits a Min pulsing behavior. The amplitude of the membrane signal decreases over time, while the fluorescence in the lumen proportionally increases. Liposome 2 does not undergo oscillations but eGFP-MinC is initially recruited to the membrane. The kymograph shows that the membrane signal continuously attenuates over time, while the lumen fluorescence intensity increases. This observation suggests that the ability of MinD to bind to the membrane decreases over time, perhaps a consequence of ATP depletion in this liposome. In Liposome 3, eGFP-MinC is only localized in the lumen and the intensity remains constant during the measurement period. This observation indicates that photobleaching is negligible.

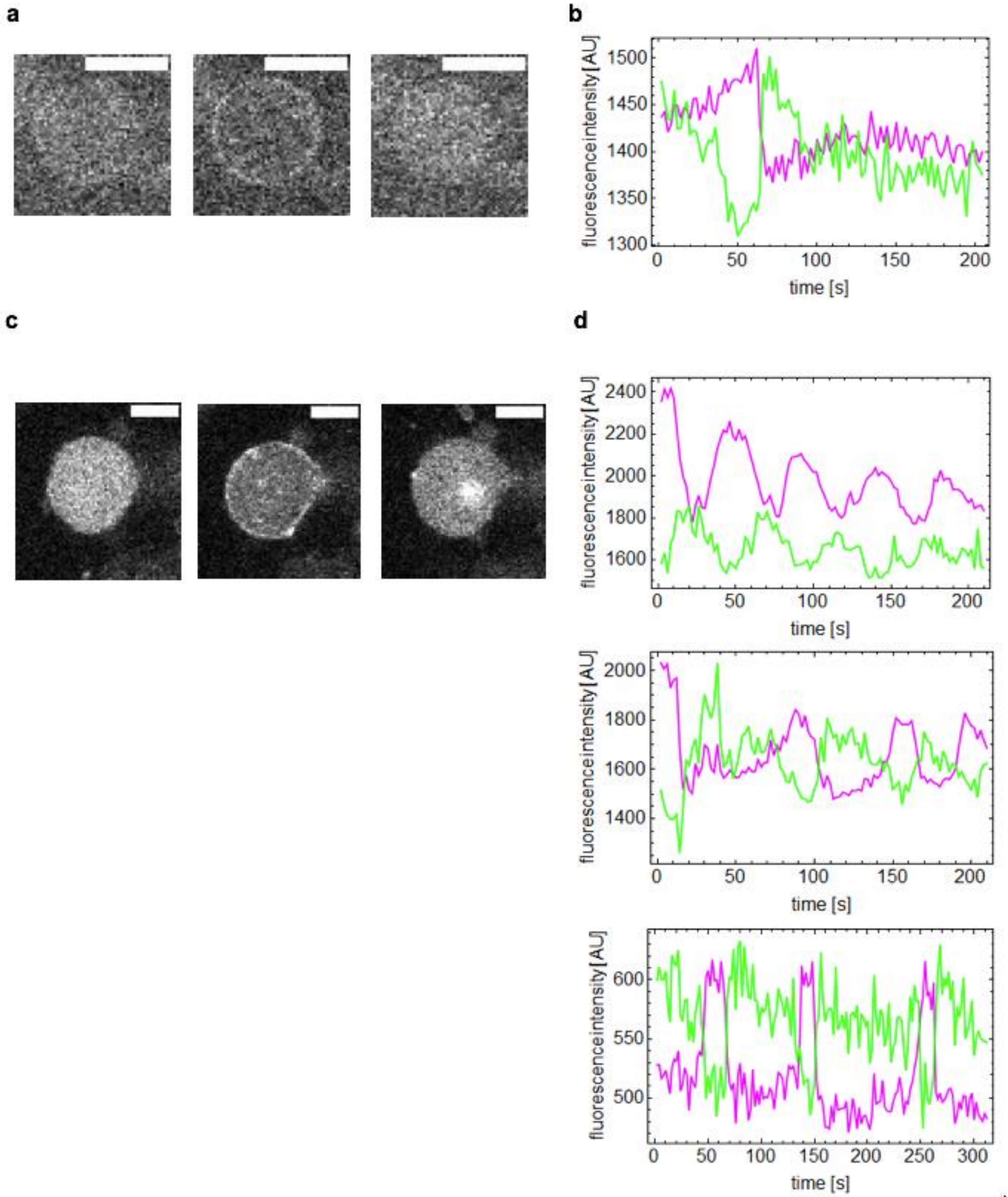

Supplementary Fig. 9: Min oscillations in liposomes using eGFP-MinD or low concentration of eGFP-MinC as a reporter. a, Time sequence images of a liposome expressing MinD/E in the presence of 0.4 µM of eGFP-MinC. **b,** Corresponding time traces of the fluorescence intensity at the membrane (magenta) and in the lumen (green). **c,** Time sequence images of a liposome expressing MinD/E in the presence of 0.2 µM of eGFP-MinD. **d,** Time traces of the fluorescence intensity at the membrane (magenta) and in the lumen (green) for three liposomes. The upper most panel corresponds to the liposome shown in **c**. Scale bars are 5 µm.

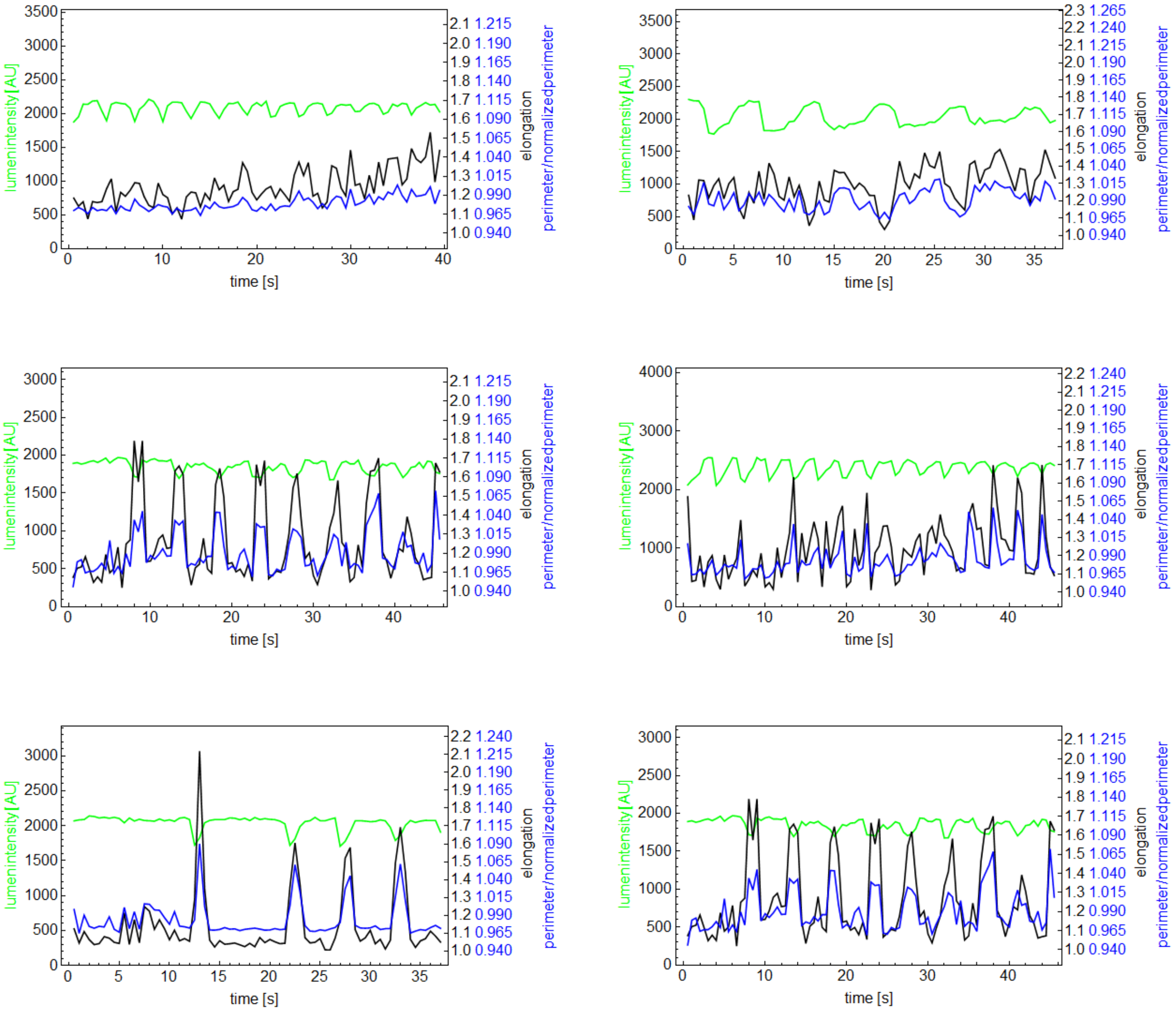

Supplementary Fig. 10: Boundary signature plots of six waving liposomes in hypertonic conditions. Lumen fluorescence intensity is colored in green, liposome elongation in black and perimeter in blue. See the Methods section for the definition of the parameters. The experimental conditions are identical as in Fig. 5. The results show that periodic MinDE-induced relocalization of eGFP-MinC from the lumen to the membrane is accompanied with an increase of liposome elongation and perimeter.

No oscillations, no membrane recruitment

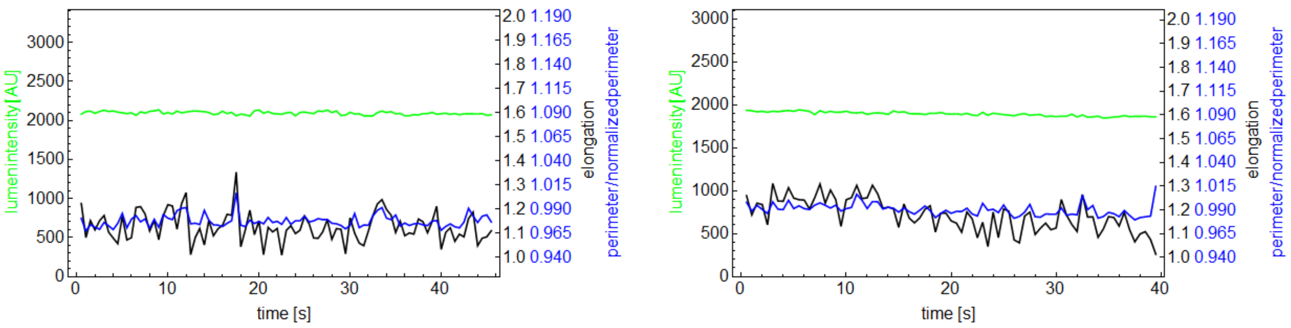

No oscillations, but membrane recruitment

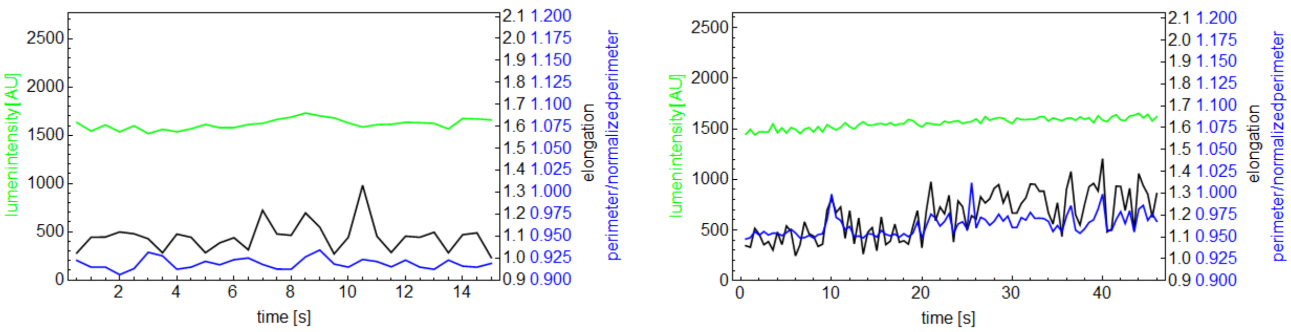

Supplementary Fig. 11. Boundary signature plots of four non-waving liposomes in hypertonic conditions. Lumen fluorescence intensity is colored in green, liposome elongation in black and perimeter in blue. See the Methods section for the definition of the parameters. The experimental conditions are identical as in Fig. 5. A clear difference in the time-course of the parameters is observed compared to Min oscillating liposomes (Supplementary Fig. 10).

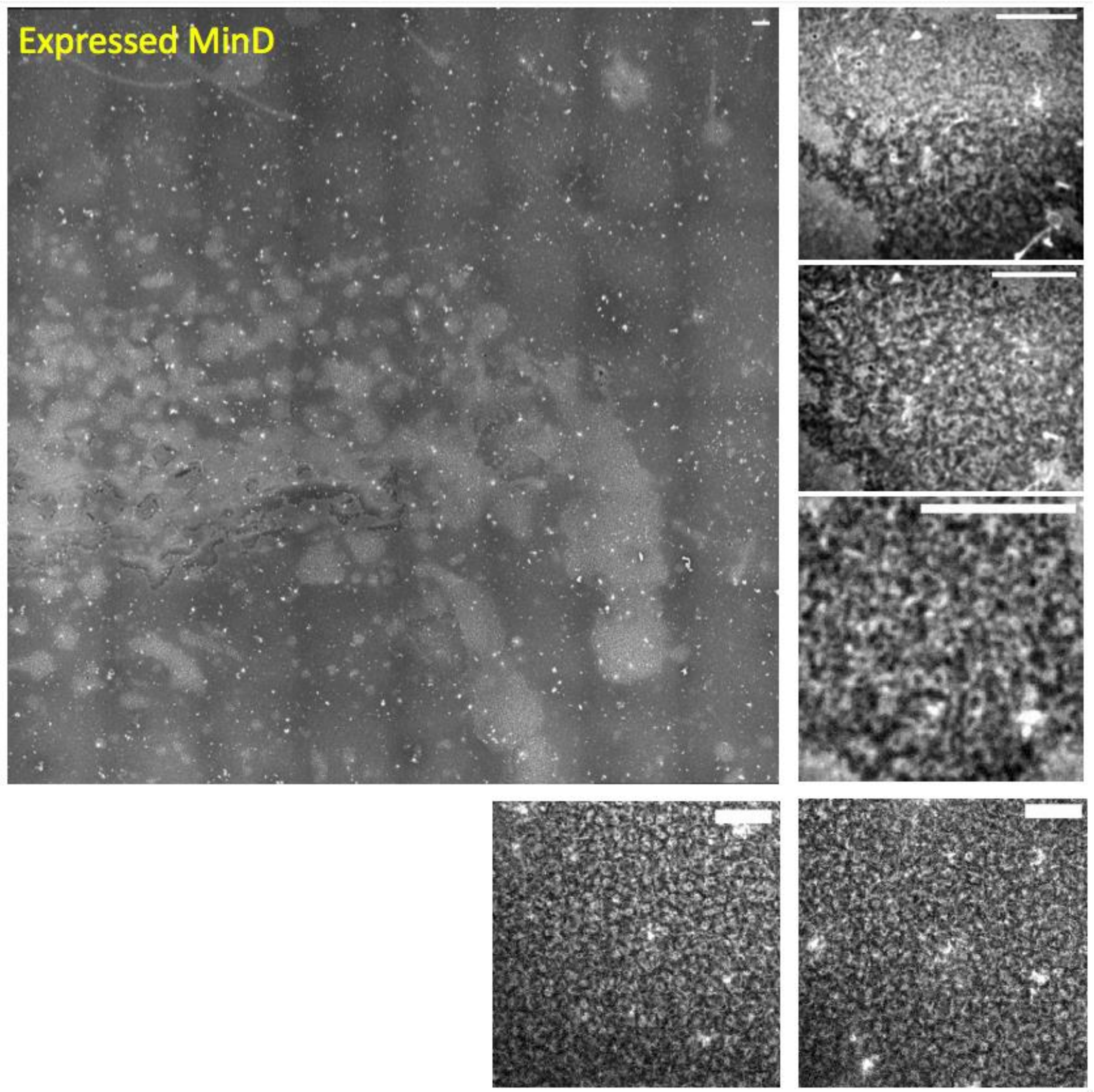

Supplementary Fig. 12: Expressed MinD was mixed with expressed FtsA, expressed TP and purified FtsZ-Alexa647, and added on top of an SLB. The conditions are as described in Fig. 6a. The large panel is a montage of 8 x 8 fields of view acquired by laser scanning confocal microscopy. The lighter areas of the SLB contain FtsZ ring-like structures. Scale bar is 20 µm. The smaller panels are zoom-in images of the SLB displaying FtsZ bundles and rings. Those images were acquired by spinning disc microscopy. Scale bars are 10 µm.

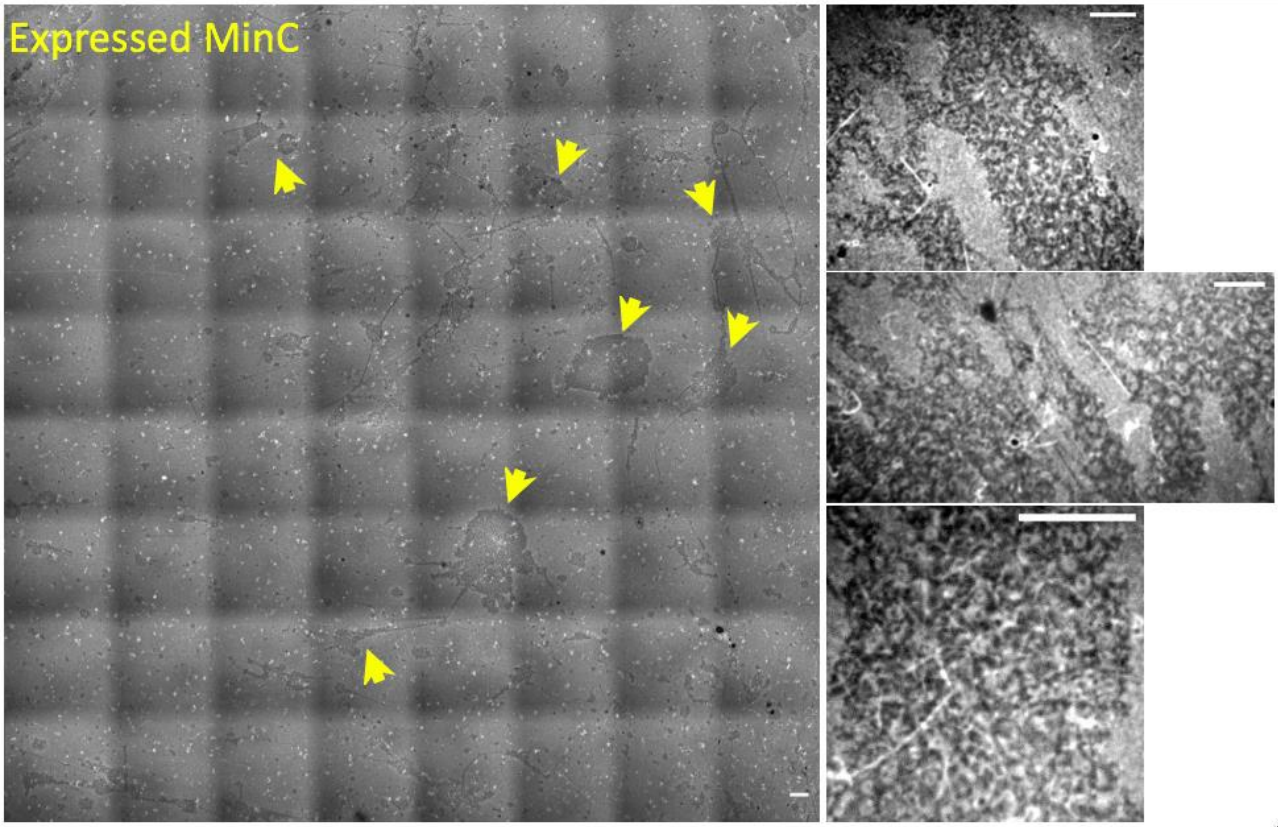

Supplementary Fig. 13: Expressed MinC was mixed with expressed FtsA, expressed TP and purified FtsZ-Alexa647, and added on top of an SLB. The conditions are as described in Fig. 6a. The large panel is a montage of 8 x 8 fields of view acquired by laser scanning confocal microscopy. The arrowheads point to areas of the SLB containing FtsZ ring-like structures. Scale bar is 20 µm. The smaller panels are zoom-in images of the SLB displaying FtsZ bundles and rings. Those images were acquired by spinning disc microscopy. Scale bars are 10 µm.

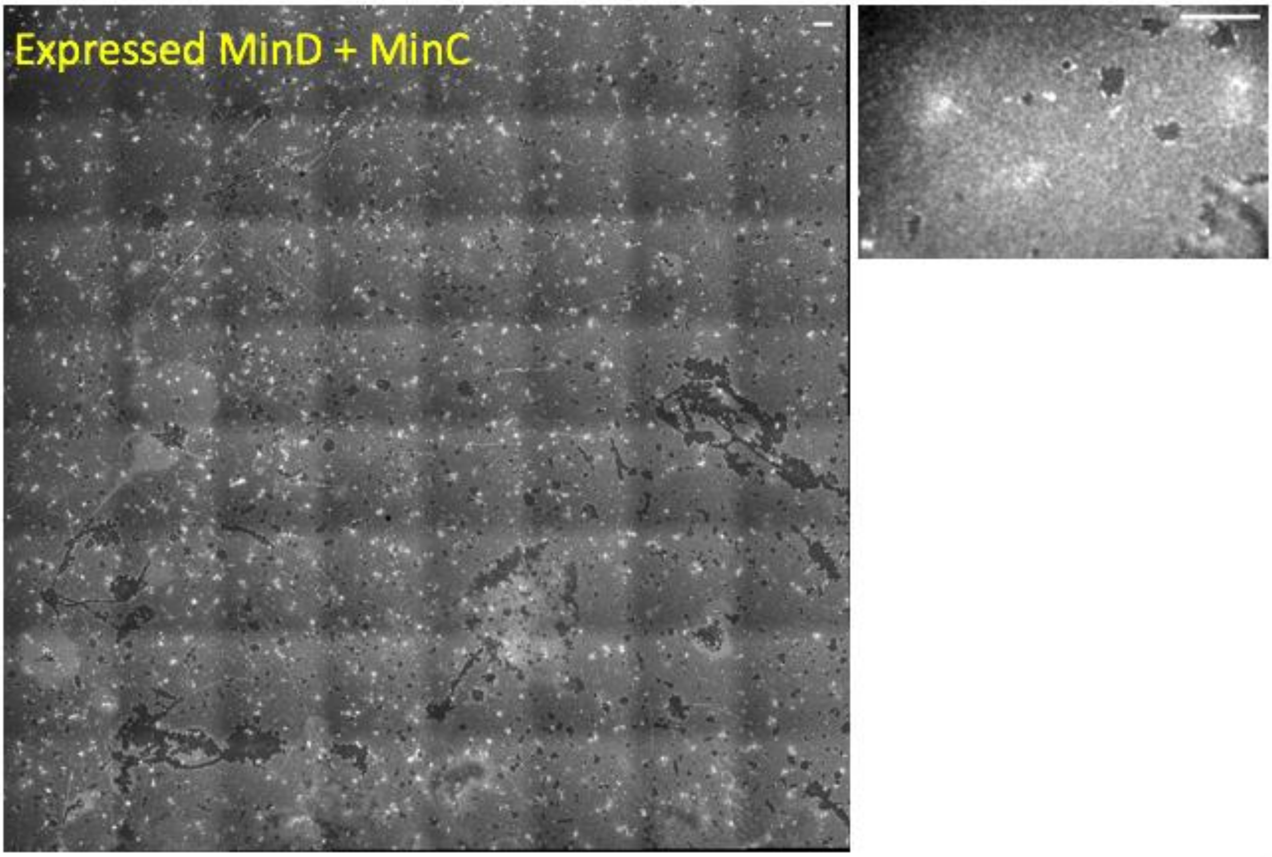

Supplementary Fig. 14: Expressed MinC and MinD were mixed with expressed FtsA and purified FtsZ-Alexa647, and added on top of an SLB. The conditions are as described in Fig. 6a. The large panel is a montage of 8 x 8 fields of view acquired by laser scanning confocal microscopy. FtsZ is recruited to the membrane but no ring-like structures appear. Scale bar is 20 µm. The smaller panel is a zoom-in image of the SLB. This image was acquired by spinning disc microscopy. Scale bar is 10 µm.

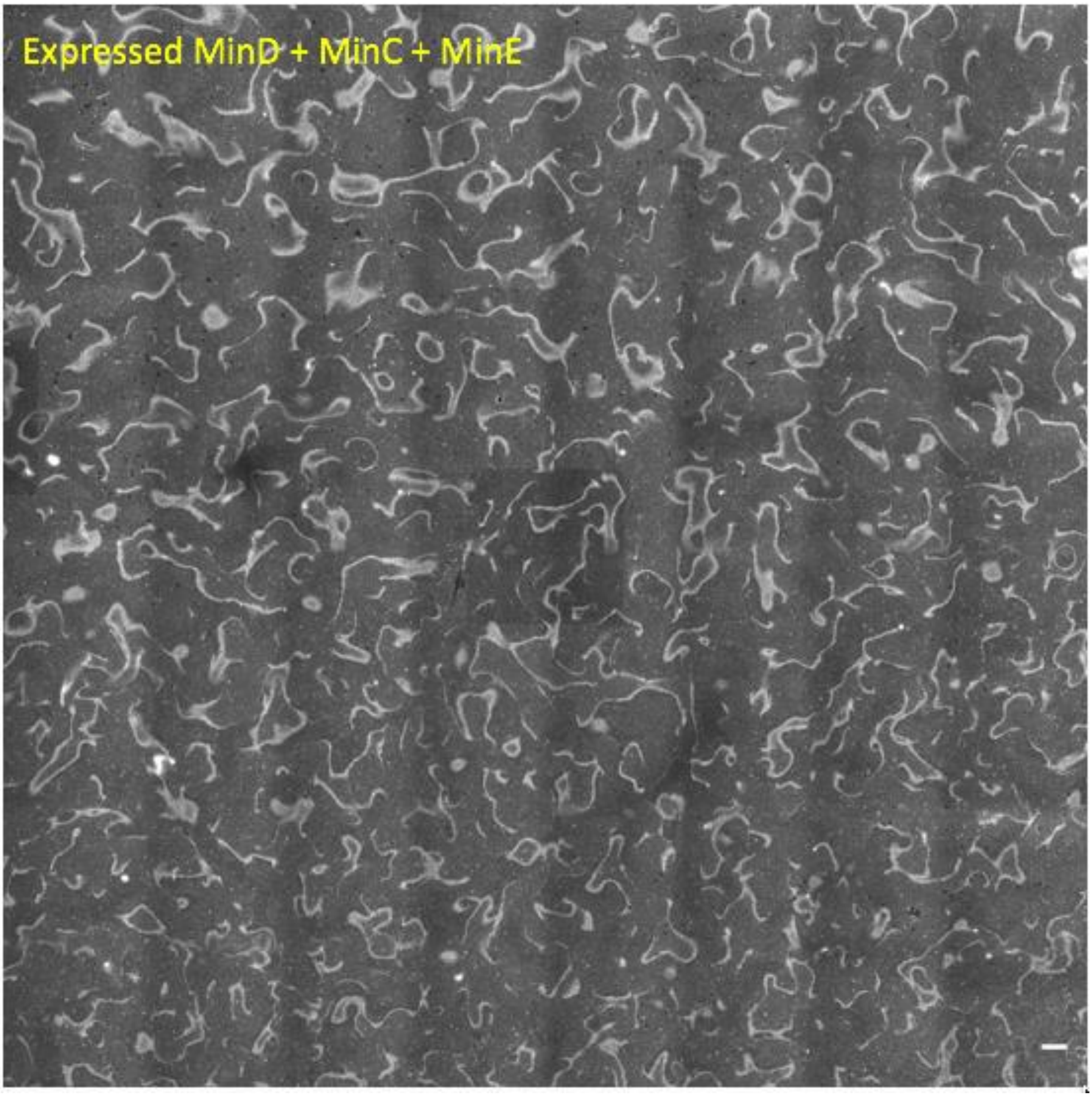

Supplementary Fig. 15: Expressed MinC and MinD/E were mixed with expressed FtsA and purified FtsZ-Alexa647, and added on top of an SLB. The conditions are as described in Fig. 6c (pattern 1). The image is a montage of 8 x 8 fields of view acquired by laser scanning confocal microscopy. FtsZ is recruited to the membrane and forms dynamic patterns. Scale bar is 20 µm.
