# Supplementary material for: De novo synthesized Min proteins drive oscillatory liposome deformation and regulate FtsA-FtsZ cytoskeletal patterns
Source: Nat Commun. 2019 Oct 31;10:4969. doi: 10.1038/s41467-019-12932-w (PMC6823393; doi:10.1038/s41467-019-12932-w)
Supplement: Supplementary file 14 — Description of Additional Supplementary Files [file 41467_2019_12932_MOESM14_ESM.pdf]

Title: Supplementary Movie 1.
Description: Planar, spiral and double spiral waves of de novo synthesized MinDE proteins on top of a supported lipid bilayer. The experimental conditions are identical as in Fig. 2. Videos of the planar, spiral and double spiral waves are played one after the other. An image of each pattern is shown in Fig. 2. Scale bar is 10 µm.

Title: Supplementary Movie 2.
Description: De novo synthesized MinDE waves change between two dynamic patterns. The experimental conditions are identical as in Fig. 2d. The video shows the same field of view after 3 h of expression and then after 4 h of expression. At 3 h one can observe travelling waves, at time 4 h the waves are standing. Scale bar is 10 µm.

Title: Supplementary Movie 3.
Description: Purified proteins in PUREfrex2.0 background on top of a supported lipid bilayer. The experimental conditions are identical as in Supplementary Fig. 7. Two videos of waves obtained with purified proteins in PUREfrex2.0 are played one after the other. The first one shows standing waves and the second video travelling waves. An image of each pattern is shown in Supplementary Fig. 7. Scale bar is 10 µm.

Title: Supplementary Movie 4.
Description: Dynamics of Min proteins in liposomes composed of PC/PG or PC/PG/PE/CL lipids. The experimental conditions are identical as in Fig. 3 and 4. PC/PG liposomes are shown first, then PC/PG/PE/CL liposomes. Not synchronized waves are present within small and large liposomes. Different dynamic behaviors can be seen. Scale bar 10 µm.

Title: Supplementary Movie 5.
Description: Pulsing, pole-to-pole and circling Min oscillations in liposomes. Videos of the three main waving patterns played in the following order: pulsing, pole-to pole and circling. The experimental conditions are identical as in Fig. 3 and 4, and PC/PG liposomes are shown. Scale bar is 10 µm.

Title: Supplementary Movie 6.
Description: Liposomes can transition from one oscillation mode to another. Three transitions are shown: From pulsing to circling, from pulsing to uncategorized, and a change in circling directionality. The experimental conditions are identical as in Fig. 3 and 4. PC/PG liposomes are shown. Scale bar is 10 µm.

Title: Supplementary Movie 7.
Description: Sole expression of MinD or MinE does not generate waves. Single expression of the minD gene leads to stable recruitment of eGFP-MinC to the membrane, whereas expression of minE leads to exclusive localization of eGFP-MinC in the lumen. The other experimental conditions are identical as in Fig. 3 and 4. PC/PG liposomes are shown. Scale bar is 10 µm.

Title: Supplementary Movie 8.
Description: Periodic liposome elongation in response to Min oscillations. The experimental conditions are identical as in Fig. 5a. No osmotic stress was externally applied. The liposome membrane dye (Texas Red) is colored in magenta. The eGFP-MinC signal is displayed in green. Scale bar is 10 µm.

Title: Supplementary Movie 9.
Description: Spatial reorganization of Min proteins induces membrane deformation in hypertonic conditions. Two videos corresponding to the two liposomes shown in Fig. 5c are played one after the other. The experimental conditions are identical as in Fig. 5c. The liposome membrane dye (Texas Red) is colored in magenta. The eGFP-MinC signal is displayed in green. Scale bar is 10 µm.

Title: Supplementary Movie 10.
Description: Surface waves of FtsZ-Alexa647 imposed by expressed MinCDE. The experimental conditions are identical as in Fig. 6c. The video shows two distinct dynamic patterns of FtsZ-Alexa647 coupled with de novo synthesized MinCDE waves. Images taken at different time points are shown in Fig. 6c. Scale bar is 10 µm.

Title: Supplementary Movie 11.
Description: Surface waves of FtsZ-Alexa647 imposed by expressed MinDE. The experimental conditions are identical as in Fig. 6d. The video shows dynamic patterns of FtsZ-Alexa647 coupled with de novo synthesized MinDE waves. A representative image is shown in Fig. 6d. Scale bar is 10 µm.
